# Bilirubin and the Yellow Players in Ferroptosis: A Delicate Balance in Human Health and Disease, with a Focus on the Liver, Cardiovascular System, and Brain

**DOI:** 10.3390/biom16091371

**Published:** 2026-09-21

**Authors:** Camilla Dalla Verde, Libor Vitek, Claudio Tiribelli, Silvia Gazzin

**Affiliations:** 1 Liver Brain Unit “Rita Moretti”, Fondazione Italiana Fegato-Onlus, Bldg. Q, AREA Science Park, ss14, Km163.5, Basovizza, 34149 Trieste, Italy; camilla.dallaverde@fegato.it (C.D.V.); ctliver@fegato.it (C.T.); 2 Department of Life Sciences, University of Trieste, 34139 Trieste, Italy; 3 4th Department of Internal Medicine, Laboratory Diagnostic, Institute of Medical Biochemistry, 1st Faculty of Medicine, General University Hospital, Charles University, 12000 Prague, Czech Republic; l.vitek@seznam.cz

**Keywords:** Nrf2, MAPK, iron, GSH/GSSG, Parkinson’s disease, Alzheimer, natural compounds, heme oxygenase, biliverdin reductase, bilirubin

## Abstract

Ferroptosis is a unique form of regulated lytic cell death driven by intracellular iron overload, excessive production of reactive oxygen species, failure of antioxidant defense systems, lipid peroxidation, and subsequent plasma membrane damage. Its initiation and progression involve complex signaling and regulatory networks. Although iron is a defining feature of ferroptosis, it also plays a central role in the heme oxygenase (HO)–biliverdin reductase (BVR) system, a cellular machinery associated with a lower prevalence of chronic diseases and reduced all-cause mortality. The HO–BVR cycle and its products biliverdin and bilirubin (together the yellow players: YPs) act as hormones and transcription factors on multiple signaling pathways, with powerful antioxidant, anti-inflammatory, and many more properties. The convergence between the two systems is suggestive of the existence of a finely tuned balance between the beneficial vs. exacerbating damage effects. Exploration of this equilibrium has just started. This review summarizes the current evidence on the interplay between ferroptosis and the HO–BVR system, in the context of liver, cardiovascular and brain diseases. We also review the therapeutic strategies aimed at modulating these pathways, considering both their potential benefits and their possible adverse consequences within the context of these integrated cellular defense and injury mechanisms.

## 1. Collection and Analysis of Literature Sources

To provide a comprehensive overview on the biomolecular possible interplay to delineate the molecular nodes potentially connecting HO1–BVR–Bilirubin (the YPs) and ferroptosis, a non-systematic step-by-step manual literature search was performed on PubMed/Google Scholar. Attention was given to studies in which the two systems were shown to regulate the same molecular target, signaling pathway, and/or metabolic process. To expand the current knowledge, both direct linking evidence and indirect mechanistic connections between the two systems were addressed, even when the relationship between YPs and ferroptosis systems was not investigated in the same paper or their connection explicitly investigated. Key terms used in the search were “unconjugated bilirubin”, “biliverdin”, “heme oxygenase”, “ferroptosis”, “iron”, “mitochondria in ferroptosis”, “glutathione and cellular redox balance”, “ferritin”, “Fen-ton reaction”, “Haber-Weiss reaction”, “pentose phosphate pathway”, “lipogenesis”, “trans sulphuration pathway”, “signaling pathways regulating the bilirubin metabolism; ferroptosis; mevalonate pathway, heme oxygenase, biliverdin reductase”, “biliverdin reductase modulators”, “heme oxygenase regulators”, “ferroptosis modulators”, “ferroptosis/iron in CVD, liver, and neurodegenerative and neurologic conditions”, “YPs modulators”, “ferroptosis modulators”, “bilirubin and ferroptosis”, “heme oxygenase and ferroptosis”, and combinations of them. The background and literature of YPs were already largely known and partly based on our published works. Each article was thoroughly reviewed and evaluated by the authors to ensure its relevance to the scope of the review. Studies were considered relevant when they provided evidence supporting a potential convergence between (i) YPs and ferroptosis; (ii) shared signaling pathways independently implicated in the two systems; or (iii) shared molecular targets and cellular processes, especially if related to iron, glutathione homeostasis, antioxidant defense, radical scavenging, or membrane protection from lipoperoxidation. A stepwise examination of the literature was done starting from the most relevant to each system. Original papers and relevant reviews were used and screened to identify additional studies to refine the manuscript in line with the strategy of the review and in line with the aim of this Special Issue.

## 2. Introduction

**Figure 1:**
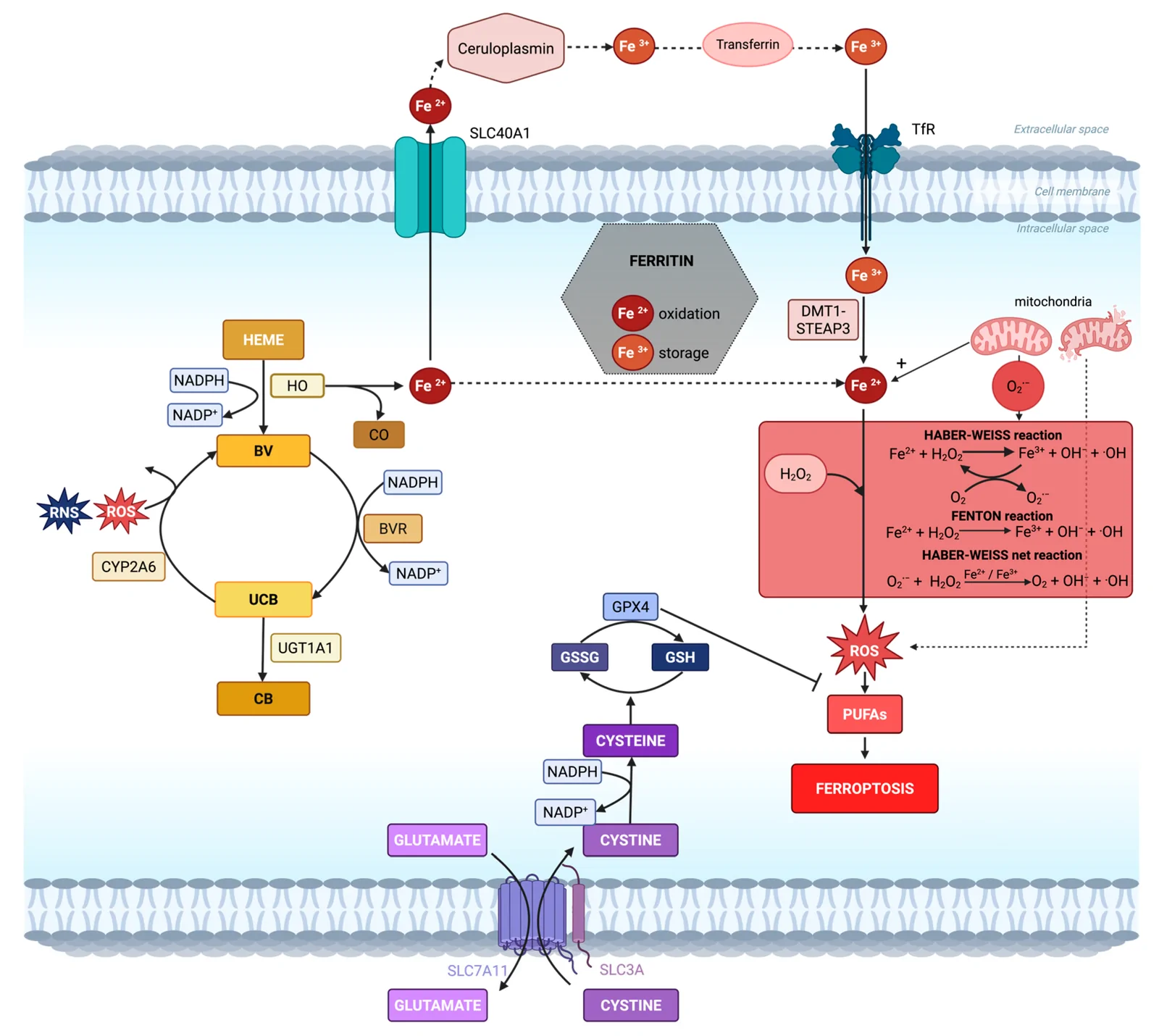
A schematic view of the YPs and ferroptosis. Solid arrows: reactions; dash arrows: same molecule for different pathways. NADPH/NADP: reduced/oxidized nicotinamide adenine dinucleotide phosphate; HO: heme oxygenase; Fe^2+^: ferrous iron; Fe^3+^: ferric iron; SLC: solute carrier protein; BV: biliverdin; BVR: biliverdin reductase; UCB: unconjugated bilirubin; UGT1A1: uridine diphosphate glucuronosyltransferase; CB: conjugated bilirubin; ROS: reactive oxygen species; NOS: nitric oxidative species; CYP: cytochrome P450 protein; TfR: transferrin receptor; GSH/GSSG: reduced/oxidized glutathione; GPX4: glutathione peroxidase 4; DMT: divalent metal transporter; PUFAs: polyunsaturated fatty acids; CO: carbon monoxide; H_2_O_2_: hydrogen peroxide; OH: hydroxyl group; OH^•^: hydroxyl radical; O_2_^−•^: superoxide. Created in BioRender. Dalla Verde, C. (2026) https://BioRender.com/ogwld9n.

This section describes the sequential action of heme oxygenase (HO) generating biliverdin (BV) which is subsequently converted into unconjugated bilirubin (UCB) by biliverdin reductase (BVR) (together yellow players—YPs; Figure 1, left). Since the late twentieth century, YPs have been recognized as one of the most potent cellular antioxidant systems. Through continuous redox cycling between BV and UCB, they can counteract a 10,000-fold excess of H_2_O_2_, acting as an antioxidant system complementary to the cytosolic reduced glutathione (GSH) (Figure 1, middle-bottom). In this way, the HO–BVR system protects membrane lipids and transmembrane proteins from oxidative damage induced by reactive oxygen species (ROS) and reactive nitrogen species (RNS) [1,2,3]. Subsequent studies have demonstrated that the HO–BVR system is widely expressed across multiple cell types and tissues, where it functions as a fundamental homeostatic network, with antioxidant and anti-inflammatory actions, modulating cell-cycle, development, glucose and lipid activities, and numerous additional biological processes that collectively support human health under physiological condition [4,5,6,7,8,9,10,11]. However, increased HO activity produces equimolar to the BV quantity of ferrous iron (Fe^2+^), the driver of ferroptosis (Figure 1, right). Ferroptosis is considered one of the most evolutionarily conserved cell death programs and contributes to physiologic development, aging, and tissue homeostasis, as well the pathogenesis of numerous human diseases [12,13,14,15,16]. Thus, the YPs–ferroptosis connections may represent a biological paradox. On one hand, increasing evidence links the activity of the YPs to a lower prevalence of chronic diseases and reduced all-cause mortality [6,8,11]. On the other hand, enhanced HO activity may increase intracellular iron availability, thereby favoring ferroptosis under conditions in which iron sequestration and antioxidant defenses are overwhelmed. Both systems engage complex and interconnected signaling networks, and considerable interest has emerged in pharmacologically targeting either or both pathways. The overlap between the two systems suggests that there is a delicate equilibrium between cytoprotection and cellular injury. Understanding how this balance is maintained—and how it is disrupted during disease—represents an emerging area of investigation. In this review, we summarize the current evidence and discuss the interplay between the HO–BVR system and ferroptosis, highlighting their mechanistic interactions and therapeutic implications in liver, cardiovascular, and brain diseases.

## 3. An Updated View on Yellow Players and Ferroptosis in Cell and Human Health and Diseases

### 3.1. The Yellow Players in Short

The well-known antioxidant activity of the YPs is based on the ability to recycle unconjugated bilirubin (UCB) to biliverdin (BV) [1,2,3] (Figure 1, left). In the first step of the process, heme oxygenase (HO), the rate-limiting enzyme of the cycle, catalyzes the oxidative cleavage of heme at the α-methene bridge carbon, released as carbon monoxide (CO), to generate biliverdin (BV), while releasing the central heme iron chelate as ferrous iron (Fe^2+^) [9,17]. BV is subsequently reduced by biliverdin reductase (BVR) to generate the lipid-soluble bile pigment unconjugated bilirubin (UCB) [5,18,19]. In these two steps, equimolar amounts of UCB (potentially antioxidant) and Fe^2+^ (pro-oxidant) are released. UCB is then conjugated (conjugate bilirubin: CB) by the UDP glucuronosyltransferase family 1 member A1 (UGT1A1), making it easily excretable from the body (urine, feces) [5,18,19]. The power of the system arises in the recycling of UCB to BV, operated by cytochrome P450 2A6 (CYP2A6), or when bilirubin acts as a direct scavenger of cellular reactive oxygen or nitrogen species (ROS, NOS). Notably, (a) this step will recycle UCB, without producing Fe^2+^; (b) due to the bilirubin’s lipophilic nature and its affinity for membrane lipids, UCB acts as an antioxidant complementary to the cytosolic reduced glutathione (GSH) [2,3,20].

### 3.2. Regulation of the YPs

**Figure 2:**
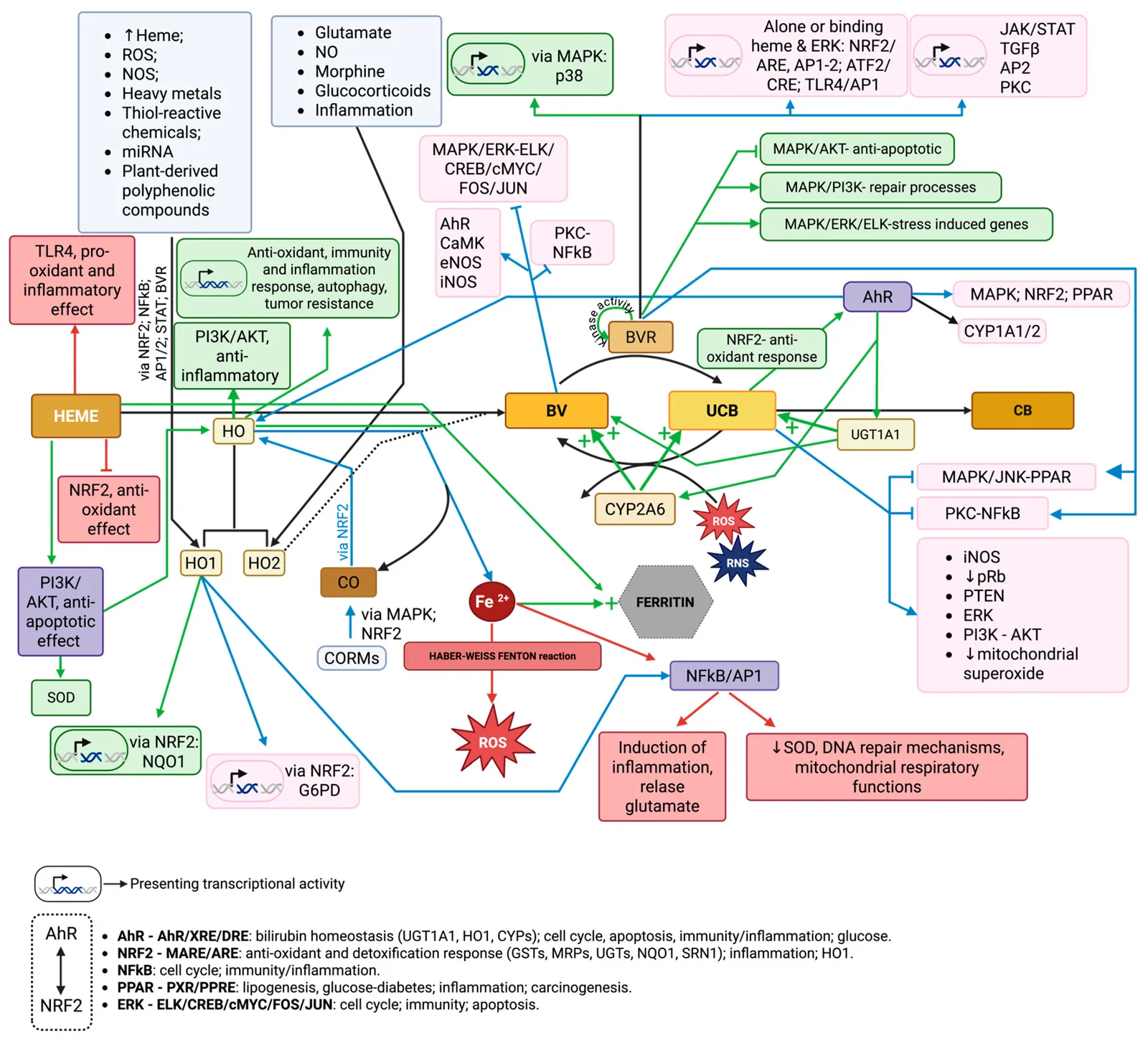
YP and signaling pathways in detail. Light blue boxes: activators; pink boxes: targets with context-related effect; red boxes: damaging pathway and effect; green boxes: protective pathway and effect; violet boxes: both targets and modulators; green arrows: protective effect; red arrows: damaging effect; blue arrows: effect is context-related; black arrows: indicate the normal pathway direction; dash line: main production of the agent. miRNA: micro ribonucleic acid; NO: nitric oxide; TLR4: toll-like receptor 4; NADPH/NADP: reduced/oxidized nicotinamide adenine dinucleotide phosphate; HO: heme oxygenase; HO1–2: heme oxygenase 1–2; Fe^2+^: ferrous iron; Fe^3+^: ferric iron; SLC: solute carrier protein; BV: biliverdin; BVR: biliverdin reductase; UCB: unconjugated bilirubin; UGT1A1: uridine diphosphate glucuronosyltransferase; CB: conjugated bilirubin; ROS: reactive oxygen species; NOS: nitric oxidative species; CYP: cytochrome P450 protein; TfR: transferrin receptor; GSH/GSSG: reduced/oxidized glutathione; GPX4: glutathione peroxidase 4; DMT: divalent metal transporter; PUFAs: polyunsaturated fatty acids; CO: carbon monoxide; MAPK: mitogen-activated protein kinase; NFkB: nuclear factor kappa-light-chain-enhancer of activated B cells; JNK: c-Jun N-terminal kinase; PPAR: peroxisome proliferator-activated receptors; ERK: extracellular signal-regulated kinase; NRF2: nuclear factor erythroid 2-related factor 2; ARE: antioxidant response element; AP1–2: adaptor protein 1–2; ATF: activating transcription factor; CRE: cAMP response element; JAK: Janus kinase; STAT: signal transducer and activator of transcription; TGF-β: transforming growth factor-β; PKC: protein kinase C; AKT: serine/threonine kinase 1; PI3K: phosphoinositide 3-kinase; ERK: extracellular signal-regulated kinase; ELK: ETS-like protein 1; AhR: aryl hydrocarbon receptor; iNOS: inducible nitric oxide synthase; pRb: phosphorylated retinoblastoma protein; NRS: reactive nitrogen species; SOD: superoxide dismutase; H_2_O_2_: hydrogen peroxide; OH: hydroxyl group; OH^•^: hydroxyl radical; G6PD: glucose-6-phosphate dehydrogenase; NQO1: NAD(P)H dehydrogenase [quinone] 1; XRE: xenobiotic response element; DRE: DNA replication-related element; MARE: Maf recognition elements; ARE: antioxidant response elements; PXR: pregnane X receptor; PPRE: peroxisome proliferator response elements; CREB: cAMP response element-binding protein; cMYC: MYC proto-oncogene; FOS: FOS proto-oncogene; JUN: JUN proto-oncogene. Created in BioRender. Dalla Verde, C. (2026) https://BioRender.com/12qfzmn.

Initially considered highly active only in the liver, YP activity has been described in multiple cells, with widespread biological actions and a readiness to respond to stimuli, expanding their pathophysiological potential [5,9,11,21,22,23,24,25,26,27,28,29,30,31,32,33,34,35,36,37,38,39] (Figure 2).

HO has two isoforms, HO1 and HO2. HO1 is considered one of the most inducible enzymes in the human body [23,40]. Its readiness to respond to a pro-oxidant environment earned it the qualification of a redox sensor [41,42,43]. HO1 responds not only to an elevated intracellular level of heme, ROS, NOS, but also heavy metals, thiol-reactive chemicals, miRNA, and a broad class of plant-derived polyphenolic compounds [10,11,44,45]. Upstream regulators of HO1 expression are NRF2 (nuclear factor erythroid 2-related factor 2), NFκβ (nuclear factor kappa-light-chain-enhancer of activated B cells), AP1,2 (activator protein), STAT (signal transducers and activators of transcription) and BVR itself [4,6,10,11,24,46]. HO1, biological actions are mediated by multiple signaling pathways, including NFkβ, AP1, and NRF2 [5,6,10]. HO1 can migrate into the nucleus, stabilizing NRF2 [47] and promoting transcriptional regulation of its several target genes, including glucose-6-phosphate dehydrogenase (G6PDH) and NQO1 (NAD(P)H quinone dehydrogenase 1), belonging, respectively, to pentose phosphate and CoQ10 (coenzyme Q10) circuits relevant in ferroptosis [47]. HO2, usually considered constitutive and responsible for maintaining the cellular BV levels required to generate UCB, supporting the cellular homeostasis role of the YP system, has recently been reported to be upregulated during inflammation and as being regulated by glutamate, nitric oxide (NO), morphine and glucocorticoids [9,10,48].

Due to its rapid reduction to UCB by BVR, BV is almost undetectable in serum and cells. Nevertheless, BV exerts regulatory functions through AhR (aryl hydrocarbon receptor, see late also UCB), protein kinase C (PKC), and Ca2+/calmodulin-dependent protein kinase (CaMK), triggering endothelial nitric oxide synthase (eNOS) phosphorylation, and inducible nitric oxide synthase (iNOS)-independent nitric oxide (NO) production [30,49,50,51].

BVR is a multifunctional protein that, in addition to catalyzing the conversion of biliverdin to bilirubin, possesses dual-specific kinase activity toward serine/threonine and tyrosine residues, thereby contributing directly to intracellular signaling [37]. Furthermore, BVR translocates from the cytosol to the nucleus in response to oxidative stress, where it regulates the transcription of genes involved in several signaling pathways, including Janus kinase/signal transducer and activator of transcription (JAK/STAT), transforming growth factor-β (TGF-β), NFκB, p38 mitogen-activated protein kinase (p38 MAPK), activator protein-2 (AP-2), PKC, and the NRF2/antioxidant response element (ARE) pathway [37,46,51,52,53,54]. By facilitating the nuclear translocation of both heme and extracellular signal-regulated kinase (ERK) into the nucleus, BVR modulates the activity of several transcriptional regulatory elements, including ARE, AP-1/AP-2, activating transcription factor-2 (ATF-2)/cyclic adenosine monophosphate (cAMP) response element (CRE), and toll-like receptor 4 (TLR4)/AP-1-responsive promoters [51]. Notably, many of these regulatory elements are also present within the promoter region of HO1 [6,46]. Importantly, the predominant adult isoform, BVRA, is highly susceptible to oxidative and nitrosative posttranslational modifications, impairing both its enzymatic and signaling activities, resulting in reduced bilirubin production and dysregulation of ERK1/2-dependent signaling [55].

The products of HO activity also exert important signaling functions. Ferrous iron (Fe^2+^), while essential for cellular metabolism, is a major trigger of Fenton chemistry, promoting the generation of reactive oxygen species (ROS) and lipid peroxides [56,57]. Consequently, increased intracellular iron stimulates de novo ferritin synthesis, which acts as a cytoprotective mechanism by sequestering excess labile iron and limiting oxidative injury [58,59].

CO, another product of HO activity, is now recognized as an endogenous gaseous signaling molecule with anti-inflammatory, anti-apoptotic, and antiproliferative properties, mediated by NRF2 and mimicked by CO-releasing molecules (CORMs) [45,60,61,62]. Notably, NRF2 activation further enhances HO1 transcription, creating an additional feedback mechanism [63].

UCB is not only a potent endogenous antioxidant but also a pleiotropic signaling molecule with immunomodulatory, antiproliferative, and cytoprotective properties [6,8,64,65,66]. UCB modulates multiple intracellular signaling pathways, including inducible nitric oxide synthase (iNOS), MAPKs, phosphatidylinositol 3-kinase/protein kinase B (PI3K/Akt), PTEN, ERK, and NF-κB signaling. It also promotes hypophosphorylation of the retinoblastoma (Rb) tumor suppressor protein, thereby influencing cell-cycle progression [6,8,64,65,66]. In addition, UCB effectively suppresses mitochondrial superoxide generation by inhibiting mitochondrial NADPH oxidase activity [31,67] and potently modulates intracellular phosphorylation cascades, mechanisms that may contribute to its proposed anticancer effects [68]. Beyond these actions, UCB serves as an endogenous agonist of the aryl hydrocarbon receptor (AhR), a ligand-activated transcription factor that regulates the expression of numerous genes, including HO1 [69], CYP1A1, CYP1A2, CYP2A6, and UGT1A1 [70]. AhR signaling is closely interconnected with both MAPK and NRF2 pathways, amplifying its biological effects [5,71]. Moreover, AhR itself is transcriptionally regulated by NRF2, further strengthening the reciprocal interaction between these signaling networks [5,72]. Finally, UCB has recently been identified as an endogenous ligand of peroxisome proliferator-activated receptor-α (PPARα), activating this nuclear receptor with an efficacy comparable to that of the pharmacological agonist fenofibrate [73].

### 3.3. Ferroptosis in Brief

Ferroptosis (Figure 1, right) is a distinct form of regulated lytic cell death characterized by unique morphological, biochemical, and metabolic features. It is driven by iron-dependent lipid peroxidation and the excessive generation of ROS, ultimately leading to plasma membrane rupture and inflammatory responses.

Intracellular iron is a key in cell homeostasis promoting cell growth, metabolism, mitochondrial oxidative phosphorylation, synthesis and releases of neuronal transmitters, myelination, etc. [71,74,75,76,77,78,79,80]. Iron is imported into the cell by the plasma membrane transferring receptor (TfR) as ferric iron (Fe^3+^) [75,81], then bound to ferritin protein for storage purposes. In ferroptosis, free Fe^3+^, may be reduced to its toxic ferrous (Fe^2+^) form by the action of divalent metal transporter 1 (DMT1)—six-transmembrane epithelial antigen of the prostate 3 (STEAP3) metalloreductase [74]. Additionally, Fe^2+^ is produced by the reduction of Fe^3+^ operated by the superoxide (O_2_•^−^) generated by cellular biological processes, including mitochondrial activity [82]. In the second step, excess Fe^2+^, in the presence of H_2_O_2_, triggers the Fenton reaction leading to the formation of hydroxide (OH^-^) and hydroxyl radical (OH^•^) responsible for the lipoperoxidation of polyunsaturated fatty acids (PUFAs) [75,83,84,85,86]). The overall reaction, known as the Haber–Weiss reaction, needs and recycle iron that works both as an electron donor or acceptor, based on its ferric or ferrous states [87,88]. Among the PUFAs, arachidonic acid (AA), docosahexaenoic acid (DHA), and eicosapentaenoic acid (EPA) are essential constituents of biological membranes, where they regulate membrane fluidity and permeability, serving as signaling molecules, and provide substrates for inflammatory mediators [85]. Their oxidative modification compromises organelle and plasma membrane integrity, ultimately causing membrane rupture and the release of damage-associated molecular patterns (DAMPs), with potential triggering of inflammation. Consequently, ferroptosis represents a mechanistic link between oxidative stress, iron dysregulation, lipid metabolism, and inflammation, and contributes to the pathogenesis of a wide range of human diseases [85,89,90,91,92,93,94].

The major endogenous defense against ferroptosis is glutathione peroxidase 4 (GPX4), that is dependent on GSH to detoxify lipid hydroperoxides formed during oxidative stress. The synthesis of GSH depends on the cystine/glutamate antiporter Xc system (Slc7a11, Slc3a2: solute carrier), mediating the entry of cystine into the cell, the rate limiting amino acid for the synthesis of GSH. The impairment of this pathway increases susceptibility to ferroptosis [16,83,95,96].

CoQ10 (ubiquinone system) and tetrahydrobiopterin (BH4) have been recently noticed as a GPX4-independent antioxidant route in ferroptosis [74]. CoQ10, the only lipid-soluble antioxidant found in humans, is an essential cofactor in mitochondrial oxidative phosphorylation producing ATP, and acts as an electron acceptor in several enzymatic reactions involving oxidation-reduction [97,98,99]. BH4 is an essential cofactor of a set of metabolic enzymes, with the ability to prevent PUFAs peroxidation by acting as a radical trapping antioxidant in lipid membranes due to its heteroatomic rings that can be oxidized (biopterin), partially reduced (dihydrobiopterin: BH2), and fully reduced (BH4) [92,100].

In addition to antioxidant defenses, the activation of endosomal sorting complex required for the transport (ESCRT-III)-dependent membrane scission machinery, essential for several fundamental cellular pathways and in remodeling and repairing damaged membranes [101], can counteract ferroptosis by shedding damaged parts of the cell membrane [102,103,104,105].

### 3.4. Regulation of the Ferroptosis Machinery

**Figure 3:**
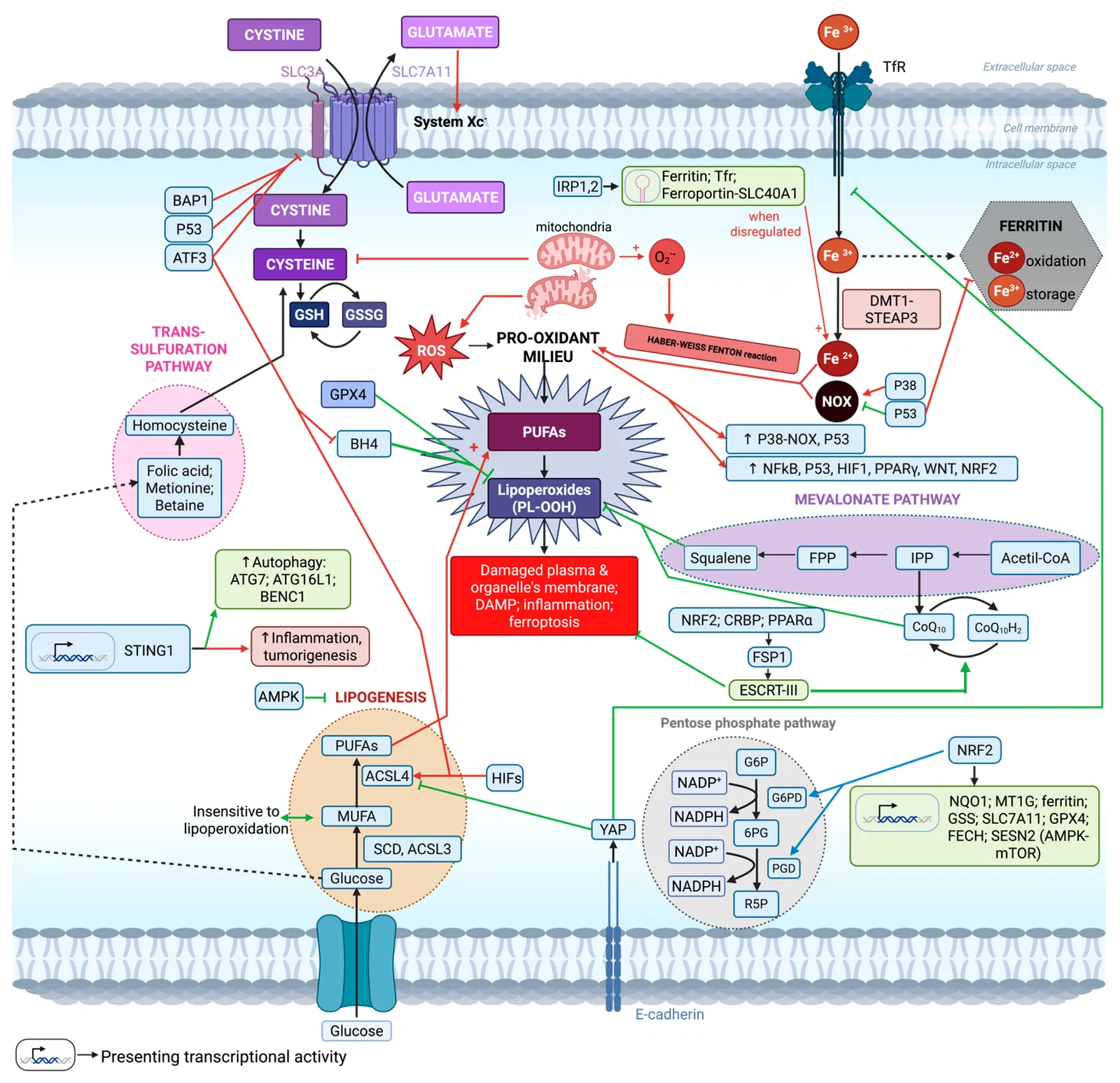
Ferroptosis and signaling pathways in detail. Light blue boxes: activators; pink boxes: agents produced; red boxes: damaging pathway and effect; green boxes: protective pathway and effect; green arrows: protective effect; red arrows: damaging effect; blue arrows: effect is context-related; black arrows: indicate the normal pathway direction; dash line: main production of the agent. NADPH/NADP: reduced/oxidized nicotinamide adenine dinucleotide phosphate; Fe^2+^: ferrous iron; Fe^3+^: ferric iron; SLC: solute carrier protein; BV: biliverdin; BVR: biliverdin reductase; UCB: unconjugated bilirubin; UGT1A1: uridine diphosphate glucuronosyltransferase; CB: conjugated bilirubin; ROS: reactive oxygen species; CYP: cytochrome P450 protein; TfR: transferrin receptor; GSH/GSSG: reduced/oxidized glutathione; GPX4: glutathione peroxidase 4; DMT: divalent metal transporter; PUFAs: polyunsaturated fatty acids; CO: carbon monoxide; NFkB: nuclear factor kappa-light-chain-enhancer of activated B cells; PPAR: peroxisome proliferator-activated receptors; NRF2: nuclear factor erythroid 2-related factor 2; ATF: activating transcription factor; BH4: tetrahydrobiopterin; BAP1: BRCA1 associated protein 1; p53: tumor protein p53; IRP: iron-regulatory protein; STING1: stimulator of interferon response cGAMP; ATG7: autophagy-related 7; ATG16L1: autophagy-related 16 like 1; BECN1: beclin1; AMPK: 5′-adenosine monophosphate-activated protein kinase; ACSL4: acyl-CoA synthetase long chain family member 4; MUFAs: monounsaturated fatty acid; HIF: hypoxia-inducible factor; SCD: stearoyl-CoA desaturase; ACSL3: acyl-CoA synthetase long chain family member 3; YAP: yes-associated protein 1; R5P: ribose 5-phosphate; PGD: phosphogluconate dehydrogenase; 6PG: 6-phosphogluconate; NOX: NADPH oxidase; H_2_O_2_: hydrogen peroxide; OH: hydroxyl group; OH^•^: hydroxyl radical; G6PD: glucose-6-phosphate dehydrogenase; G6P: glucose-6 phosphate; ESCRT III: endosomal sorting complex required for transport III; FSP1: ferroptosis suppressor protein 1; CRBP: cellular retinol-binding protein; CoQ_10_: coenzyme Q_10_; CoQ_10_H_2_: ubiquinol; IPP: isopentenyl pyrophosphate; FPP: farnesyl pyrophosphate; WNT: wingless; STEAP3: six-transmembrane epithelial antigen of the prostate 3; NQO1: NAD(P)H dehydrogenase [quinone] 1; MT1G: metallothionein 1G; GSS: glutathione synthetase; FECH: ferrochelatase; SESN2: sestrin 2; MTOR: mechanistic target of rapamycin kinase; Xc system: cystine-glutamate transport system. Created in BioRender. Dalla Verde, C. (2026) https://BioRender.com/n37uxpq.

Ferroptosis is carried out through sophisticated metabolic and signaling and mechanisms that regulate intracellular iron homeostasis, lipid metabolism, redox balance, membrane repair, and inflammatory response (Figure 3) [12,106,107].

Iron homeostasis is under the control of the iron-regulatory proteins IRP1 and IRP2, which engage in post-transcriptional regulation of IRE (iron-responsive elements) genes involved in intracellular iron storage/release and import/export (e.g., ferritin, TfR, ferroportin-SLC40a1) [108,109]. Dysregulation of this network enlarges the labile iron pool, enhancing Haber–Weiss–Fenton chemistry, ROS production, and lipid peroxidation. Notably, not only iron, but also other transition metals may act in the same way when generating ROS, thus facilitating ferroptosis, which may be beneficial in cancer therapy [110]. Iron and superoxide (O_2_^•−^) are key in the Haber–Weiss reaction. One source of O_2_^•−^·— are mitochondria [82], which plays a major role in cysteine deprivation-induced ferroptosis, leading to altered mitochondrial morphology, a landmark of ferroptosis [111,112,113]. Moreover, mitochondria have been identified as having regulatory roles in ferroptosis playing at multiple levels (amino acid metabolism, lipid metabolism, mitochondrial labile iron pool and ferritin transporter, and energetic metabolism) and contributing to cancer, cardiovascular diseases, neurodegenerative diseases, and more, as deeply reviewed in [112]. On the other side, bilirubin binds and stabilizes mitochondria membranes at physiologic concentrations [114]. Mitochondrial dysfunction and mitochondrial fatty acid synthesis also produce ROS, promoting lipid peroxidation [111,115,116,117,118]. Oxidative stress resulting from the imbalance between the production of ROS and the availability of antioxidants or free radical scavengers further activates an abundance of transcription factors and pro-inflammatory genes, including NFκB, p53 (transcription factor tumor protein), HIF1α (hypoxia-inducible factor), PPARγ, β-catenin/Wnt, and NRF2 [83]. Ferroptosis can also start as a consequence of blocking antioxidant defenses [74,119]. In addition to its import mediated by the Xc system, cysteine may be produced by the transsulfuration pathway, which is negatively regulated by cysteinyl-tRNA synthetase 1 (CARS1) [120]. Elevated extracellular glutamate may inhibit cystine uptake through the Xc system, erasing the GPX4 antiferroptotic protection capacity [12,121,122]. Activation of the transcription factor 3 (ATF3) transcriptionally suppresses SLC7A11, similarly reducing the levels of GSH and GPX4 but increasing the acyl-CoA synthetase long-chain family member 4 (ACSL4). ACSL4 upregulation promotes fatty acid metabolism and exacerbates lipid oxidation [86,123]. BRCA1-associated protein 1 (Bap1) sensitizes cells to ferroptosis by suppressing Slc7a11 synthesis [16], while the tumor suppressor gene p53 also transcriptionally inhibits SLC7A11 expression [124,125], with a simultaneous decrease in the expression of ferritin (FTH1: ferritin heavy polypeptide 1), thereby increasing the intracellular labile iron pool. Activation of the stimulator of interferon response CGAMP interactor 1 (STING1, also known as TMEM173) pathway by DNA damage signals plays a dual role in promoting or inhibiting ferroptosis (by triggering autophagy-related protein (ATG) 5, 7 and 16L1 and beclin (BECN) 1 [94,126,127,128,129] depending on its gene targets or binding proteins [130]. It also induces inflammation-related cell death [131,132,133]. At least in cancer, ROS increase and lipid peroxidation correlate with the activation of MAPK14 (best known as p38), which up-regulates the expression of NOX (nicotinamide adenine dinucleotide phosphate (NADPH) oxidase) protein family and negatively correlates with the transcription factor tumor protein p53 [134,135]. The NOX family is historically known as the first identified example of a system that generates ROS not as a byproduct, but rather as the primary function of the enzyme system in phagocytosis [136]. They may thus contribute to enhancing redox stress and ferroptosis.

Lipid metabolism represents another major regulatory node. ACSL4 inhibition plays a key role in promoting ferroptosis by shifting the production from PUFAs to monounsaturated fatty acids (MUFAs), insensitive to lipoperoxidation [85,137,138,139]. MUFA production is favored by the action of stearoyl-CoA desaturase (SCD) [140] and acyl CoA synthetase long-chain family member 3 (ACSL3) [85,140,141]. Likewise, AMPK mitigates ferroptosis by reducing lipogenesis, triggering protective programs against ferroptosis associated with energy failure that involves impaired biosynthesis of PUFAs [90,116,142,143]. During hypoxia (in carcinoma), hypoxia-inducible factors (HIFs) are activated, further remodeling lipid metabolism, enriching cellular membranes with oxidizable PUFAs and thereby sensitizing cells to ferroptosis [144].

GSH belongs to thiol disulfide systems. This system includes multiple cellular organic compounds containing a sulfhydryl functional group (-SH; e.g., cysteine, thioredoxin, taurine, homocysteine, lipoic acid, CoA, and albumin). The sulfidic group is prone to accept (and return) the excess electrons present in pro-oxidant milieu, forming disulphide bonds and playing a crucial role as a redox buffering system, including protecting from lipoperoxidation [145]. UCB also acts as a sacrificial anode when it is recycled to BV (Figure 1) by scavenging ROS. Notably, the thiol disulfide systems and UCB compete and/or synergize in the cellular defense against ROS [29,146,147,148], adding relevance to YPs as synergic to GSH cytoplasmatic protectors towards oxidation [2]. The same synergy possibly applies to alpha-tocopherol [1,149], a potent peroxyl radical scavenger in ferroptosis [150]. Moreover, albumin is not only a major thiol disulfide extracellular antioxidant but a ligand for UCB, determining the fraction of the pigment available for the biological function discussed in this paper (free bilirubin) [6,11,148,151,152].

Several GPX4-independent antioxidant systems have recently emerged as major suppressors of ferroptosis. Ferroptosis suppressor protein 1 (FSP1, also known as apoptosis-inducing factor mitochondria-associated 2—AIFM2), as well as p53-responsive gene 3 (PRG3), has been shown to modulate lipoperoxidation through an ESCRT-III-dependent membrane repair mechanism [153], as well as CoQ10 [99]. CoQ10 is also controlled by the mevalonate pathway, converting Acetyl-CoA to isopentenyl pyrophosphate (IPP) and cholesterol precursors, including squalene [154]. The synthesis of squalene and its release in the endoplasmic reticulum protects the cell against lipid peroxidation, resulting in cell survival [155]. FSP1 also reduces ubiquinone to ubiquinol thanks to its NADH: ubiquinone reductase activity, in turn reducing lipid radicals terminating lipid peroxidation [156,157], finally suppressing ferroptosis [99,157]. Notably, FSP1 is transcriptionally regulated by both NRF2 and PPARα, linking antioxidant defenses with lipid metabolism [158,159,160].

The Hippo signaling pathway (YAP and, in certain cancers, TAZ) involved physiological functions such as cell growth, shape, and dimension, and it also modulates ferroptosis. YAP is sensitive to E-cadherin-mediated cell–cell contact. After migrating into nuclei, YAP regulates the transcription of several ferroptosis players, among them ACSL4 and NOX, thus acting as a suppressor for ferroptosis [161,162]. At least in cancer, YAP/TAZ have been shown to negatively (lower YAP/TAZ, higher BH4) regulate biopterin (BH4) metabolism (low YAP/TAZ, high BH4), thereby influencing another GPX4-independent antioxidant system [163].

Among all protective pathways, the activation of NRF2 represents the master transcriptional response against ferroptosis [119,164]. The release of NRF2 from KEAP1 (Kelch-like ECH associated protein 1), allows NRF2 to translocate into the nucleus and activate a broad antioxidant and cytoprotective transcriptional program such as NQO1, metallothionein 1G (MT1G), glutathione synthetase (GSS), sestrin 2 (SESN2), and ferrochelatase (FECH), as well as SLC7A11, GPX4, ferritin, ferroportin (SLC40A1), FSP1, G6PDH and pyruvate decarboxylase, belonging to the pentose phosphate pathway, relevant because of the regeneration of NADPH, which is largely consumed in ferroptosis, and HO1, the last playing a dual role in ferroptosis [164,165,166,167,168,169,170,171,172,173,174].

## 4. YPs and Ferroptosis in Liver, Cardiovascular System and Brain

### 4.1. Ferroptosis and Liver Diseases

Recent evidence has shown ferroptosis as a key mechanism in the pathogenesis of multiple liver diseases, particularly metabolic dysfunction-associated steatotic liver disease (MASLD) and metabolic dysfunction-associated steatohepatitis (MASH) [175]. The process is driven by the accumulation of iron-catalyzed lipid hydroperoxides in PUFAs-containing membrane phospholipids when antioxidant defense systems, particularly the glutathione pathway, become overwhelmed. Human studies demonstrate an increase in liver lipid peroxidation products, such as malondialdehyde, 4-hydroxynonenal and oxidized phospholipids, in MASLD/MASH, supporting the presence of ferroptotic stress [176]. Beyond steatohepatitis, ferroptosis contributes to acute liver injury, fibrosis, cirrhosis, and hepatocellular carcinoma (HCC). Importantly, ferroptosis plays a dual role in liver disease: inhibition of ferroptosis can attenuate hepatocyte injury, inflammation, and fibrosis, while its induction appears a promising strategy to suppress ferroptosis-sensitive HCC cells [176]. Together, ferroptosis has emerged as both a mechanistic driver of liver disease progression and a promising therapeutic target, which warrants further translational and clinical investigation [175]. Glutathione (GSH), a tripeptide particularly concentrated in the liver, is the most important thiol reducing agent involved in the modulation of redox processes [177] and also a central antiferroptotic defense in the liver [178,179]. In liver cells, the GPX4 axis is one of the main systems that prevents lipid peroxidation, because GSH serves as the essential cofactor for GPX4, which reduces toxic lipid hydroperoxides. When GSH is depleted, GPX4 activity falls, lipid peroxides accumulate, and ferroptosis is more likely to occur [178,179]. Its intracellular content is regulated, among other mechanisms, by γ-glutamyl transferase (GGT), which regenerates GSH that leaks and escapes from the liver cells [180]. Elevated GGT is a biochemical hallmark of MASLD reflecting the depletion of intracellular GSH in steatotic liver cells (as well as other forms of chronic liver disease) exposed to increased lipoperoxidation [177,181]. Bilirubin acts as a complementary antioxidant to GSH. GSH is the main water-soluble antioxidant and primarily protects proteins in the aqueous phase, while bilirubin is lipophilic and is more effective in protecting membrane lipids from oxidation [2]. Thus, bilirubin can reduce the oxidative burden that would otherwise consume GSH. In addition, bilirubin can influence GSH handling more directly in the liver. In experimental studies, bilirubin was shown to inhibit biliary secretion of GSH in a dose-dependent manner [182,183]. As a result, serum bilirubin is negatively associated with GGT activity [184], most likely as a result of the amelioration of increased oxidative stress in hepatocytes suffering from liver diseases. Hence, a higher bilirubin concentration appears to be associated with a lower functional demand for redox compensation, while an elevated GGT activity reflects a higher oxidative burden. Together, bilirubin interacts with GSH metabolism by acting as a complementary antioxidant, reducing lipid peroxidation stress and, in some settings, altering glutathione secretion and GST-related detoxification pathways. Although elevated serum bilirubin concentrations are generally considered an ominous sign of underlying liver disease, the data discussed suggest that mildly increased bilirubin can counteract ferroptotic damage in the liver by acting as a potent cellular antioxidant [1] and perhaps an iron chelator, as experimentally described in transplanted pancreatic islets [184].

### 4.2. Ferroptosis and Cardiovascular Diseases

Ferroptosis is also associated with cardiovascular diseases (CVD), including atherosclerosis, myocardial infarction, ischemia–reperfusion injury, heart failure, cardiomyopathy, myocarditis, arrhythmia, aneurysm, and valve calcification [185]. Although the evidence is mostly indirect, bilirubin appears to also affect ferroptosis-related processes in CVD. Similarly, as in liver diseases, bilirubin, as a strong antioxidant, could reduce oxidative stress and lipid peroxidation, which are the central drivers of ferroptosis in CVD. Therefore, it is likely that bilirubin modulates the ferroptotic process by improving the redox environment rather than directly blocking the ferroptotic pathway itself [186]. The main mechanisms by which bilirubin could affect ferroptosis in CVD include the following:

- Direct scavenging of reactive oxygen species and peroxyl radicals with inhibition of lipid peroxidation, which would reduce the oxidative burden that drives ferroptosis [187].
- Bilirubin can protect endothelial cells from oxidative injury, which is relevant because endothelial dysfunction is an early event in atherosclerosis and a ferroptosis-sensitive process [188].
- Suppression of NADPH oxidase-derived superoxide generation resulting in a decrease in downstream lipid oxidation [67] (PS e via NOS).
- Reduction in vascular inflammation and leukocyte adhesion, which could indirectly limit ferroptosis-promoting inflammatory stress in the vessel wall [189].
- Improvement of GSH metabolism in the cardiovascular system. GGT is increasingly viewed as a CVD risk marker rather than just a liver enzyme. Higher GGT activity is associated with an increased risk of coronary heart disease, stroke, heart failure, arrhythmias, and CVD mortality [190]. GGT metabolizes extracellular glutathione to produce cysteine and cysteinyl glycine, which can promote iron-dependent LDL oxidation; GGT activity has also been found in atherosclerotic plaques, supporting a potential role in local vascular injury [191]. Importantly, recent clinical studies showed a negative association between bilirubin and GGT, with low bilirubin and high GGT jointly reflecting a more oxidative cardiovascular risk profile [184,192].

Therefore, bilirubin can modulate ferroptosis in CVD primarily by reducing ROS generation, inhibiting lipid peroxidation, preserving endothelial function, and damping inflammatory vascular injury.

### 4.3. Ferroptosis and the Brain

Accumulating evidence has identified ferroptosis as a key pathogenic mechanism in a wide range of neurological disorders, including ischemic stroke (IS), Huntington’s disease (HD) [193], multiple sclerosis (MS), amyotrophic lateral sclerosis (ALS) [194,195,196], and neurodegenerative diseases such as Alzheimer’s disease (AD) and Parkinson’s disease (PD) [196]. A common feature across these conditions is the abnormal accumulation of iron within affected brain regions, where iron deposits are frequently observed at sites of neurodegeneration or tissue injury (HD: [193]; AD and PD: [196,197,198]; ALS: [194,195,196], mainly resulting in an exacerbation of the damage [196]. Different mechanisms have been proposed, depending on the disease and experimental models.

#### 4.3.1. Parkinson’s Disease

Postmortem studies of patients with PD consistently demonstrate increased iron accumulation, elevated levels of ROS and lipid peroxidation products, together with depletion of GSH and reduced GPX4 expression or activity, largely associated with dysregulation of the Nrf2 antioxidant pathway [12,199,200,201,202,203,204]. Two major contributors to oxidative stress in PD are α-synuclein (α-syn), which forms the characteristic Lewy body inclusions, and cytosolic dopamine (DA), whose oxidation generates large amounts of ROS [197,205]. These alterations establish a pro-oxidative environment that favors ferroptotic cell death. Oxidation of dopamine, either enzymatically or through ROS-mediated reactions, also leads to the formation of neuromelanin (NM). Owing to its high ability to chelate transition metal, especially Fe^2^+, NM is usually protective in neurons. However, experimental models suggest that excessive intracellular accumulation of NM parallels PD progression, possibly by inducing α-synuclein expression and aggregation, thereby establishing a self-perpetuating cycle of oxidative stress, iron dysregulation, and neurodegeneration [206]. Moreover, α-syn possesses iron-responsive elements (IRE) in its mRNA’s 5′ untranslated region, making its expression directly under control of ferroptotic machinery. Mutations in the transferrin receptor 2 (TfR2) have been suspected to reduce iron uptake, strengthening the link in between iron, ferroptosis and this neurodegenerative condition [207].

HO1 is markedly upregulated in the substantia nigra as well as in dopaminergic neurons, microglia, and astrocytes in PD brains [208,209,210,211,212,213]. HO1 activation exerts both potentially detrimental and protective effects: while the released iron may enhance ferroptosis, BV and particularly UCB possess potent antioxidant and anti-inflammatory properties that may counteract oxidative injury. The strict interplay between dopamine (DA) and HO1/YPs does not stop here. DA up-regulates HO1 in vitro [214], whereas UCB inhibits several aspects of DA metabolism, including tyrosine uptake; cAMP-stimulated DA synthesis—but not basal production, DA reuptake and cellular vesicular storage [215,216,217,218]. However, many of these studies employed bilirubin concentrations (70–140 μM) that exceed the physiological range and may themselves exert neurotoxic effects, limiting their translational relevance. Nevertheless, the neuroprotective potential of UCB has been consistently demonstrated in experimental models of PD [219], and more recently supported by a population-based study. Analysis of the National Health and Nutrition Examination Survey (NHANES), including 25,637 participants enrolled between 1999 and 2018, revealed a lower prevalence of PD among individuals with Gilbert syndrome, a condition characterized by chronically mild unconjugated hyperbilirubinemia and a systemic antioxidant and anti-inflammatory phenotype [220]. Furthermore, genetic polymorphisms of the HO gene associated with reduced HO1 activity and consequently lower BV and UCB production have been shown to correlate with the early onset of PD (reviewed in [7]). The overall impact of the YP pathway in the ferroptotic enhancement of neurodegeneration is therefore likely to depend on the balance between iron release and bilirubin-mediated cytoprotection, a relationship that warrants further mechanistic investigation.

#### 4.3.2. Alzheimer Disease

In AD, iron accumulation not only enhances ferroptosis progression but also correlates with β-amyloid (Aβ) load in affected brain regions [198,221,222]. In turn, Aβ preferentially targets PUFA-rich membrane phospholipids and disrupts intracellular calcium homeostasis, leading to mitochondrial dysfunction, excessive ROS generation, and enhanced lipid peroxidation. Brains from patients with AD exhibit decreased activity of glutamine synthetase (GS), decreased GSH content, and increased lipid peroxidation, reflecting impaired antioxidant defenses and increased susceptibility to ferroptosis (reviewed in [198,221,222]). The Fenton/Haber–Weiss reaction plays a significant role in AD, since hydroxyl radicals may (a) convert soluble fibrinogen into the insoluble fibrin-like aggregate observed in AD patients; (b) activate signal pathways and transcription factors shared with YPs (e.g., ERK1/2, JNK, PI3K, p38-MAPK [223]).

The role of YPs in AD has been extensively investigated. Both HO1 and BVRA are increased in the brain of patients. However, the highly oxidative environment characteristic of AD promotes oxidative modification of BVRA, impairing its enzymatic activity. As a result, BV (and iron) but not UCB are produced, enhancing ferroptosis and simultaneously weakening endogenous antioxidant defenses [55,224]. Experimental studies further demonstrate that impaired BVRA activity promotes β-secretase 1 (BACE1) activation, increases Aβ deposition, and induces neuronal insulin resistance through down-regulation of the insulin receptor [225,226]. Interestingly, insulin signaling and the YP pathway appear to regulate one another [5], with bilirubin increasing the expression of the insulin receptor, SREBP1 (sterol regulatory element-binding protein), and PPARγ [227]. These reciprocal interactions further support a mechanistic link between bilirubin metabolism, insulin signaling, ferroptosis, and AD pathogenesis [228]. Collectively, these findings suggest that dysfunction of the YP pathway contributes to AD progression by disrupting the balance between iron release and bilirubin-mediated antioxidant defense. Impaired bilirubin production, together with increased intracellular iron availability, may enhance ferroptotic neuronal death, whereas preservation of BVRA activity and bilirubin generation could represent a novel therapeutic strategy to limit oxidative damage and neurodegeneration in AD.

#### 4.3.3. Ischemic Stroke

Ferroptosis has emerged as a major contributor to neuronal injury following ischemic stroke (IS). In the transient middle cerebral artery occlusion model, ischemia induces intracellular iron overload, largely as a consequence of the down-regulation of ferroportin-1 (FPN1), transporting Fe^2+^ out of the cell [229], thus promoting ROS generation, lipid peroxidation, and ultimately ferroptotic cell death. Consistent with these findings, experimental cerebral ischemia–reperfusion models of IS demonstrated iron cellular overload, ROS, malondialdehyde (MDA) increase, and GSH and GPX4 decrease with increased cell death, accompanied by the increase in HIF1. The experimental overexpression of HIF1α (by vectors) attenuates cell death by increasing FPN1, GSH and GPX4. In the model, HO1 is also upregulated. This suggests the activation of the HIF1α pathway as a potential therapeutic strategy for limiting ischemia-induced ferroptosis. Notably, NRF2 acts as a central regulator of all the aforementioned mechanisms [230,231].

#### 4.3.4. Huntington’s Disease

Increasing evidence from both clinical studies and experimental models indicates that dysregulated ferroptosis contributes to the pathogenesis of HD. Hallmarks of ferroptotic dysfunction in HD include depletion of GSH, reduced GPX activity [232,233,234], and upregulation of TfR1, leading to increased cellular iron uptake. In line, genetic silencing of ACSL4 attenuates neuronal injury in experimental HD models [235], further supporting a pathogenic role for ferroptotic signaling. The heme oxygenase YP pathway may also contribute to the regulation of ferroptosis in HD. HO1 induction in vivo models of HD resulted in protection by reducing lipid oxidation, oxidative stress and inflammation [236]. Although the precise mechanisms remain to be elucidated, these findings suggest a theoretical link in between ferroptosis and YPs, which needs further experimental investigation.

#### 4.3.5. Multiple Sclerosis

MS is characterized by the destruction of the myelin sheath surrounding axons. Myelin, produced by oligodendrocytes, is highly enriched in lipids, particularly PUFAs, the primary target of ferroptosis [237]. Consequently, it is not surprising that the presence of MDA has been consistently reported in patients with MS [237,238]. Experimental evidence further supports the involvement of ferroptosis in MS pathogenesis. In the experimental autoimmune encephalomyelitis (EAE) model of MS, disease severity is associated with a higher expression of ferroptosis-related markers; TFR1, ACSL4 and NCOA4, and with a lower expression of GPX4, xCT and FTH1 (coding for the heavy chain of ferritin) [239]. On the other hand, pharmacological inhibition of ferroptosis with ferrostatin-1 (Fer-1) significantly reduces lipid peroxidation attenuates and clinical disease severity, providing strong evidence that ferroptosis contributes to demyelination and neurological dysfunction [239]. The protective effects of UCB on the EAE model are documented. Administration of UCB has been shown to alleviate clinical manifestations of EAE while reducing oxidative stress, lipid peroxidation, and neuroinflammation [240,241,242]. The exact molecular mechanisms linking YP metabolism to ferroptosis in MS remain largely unexplored and need empirical evaluation.

## 5. The Bidirectional Interplay Between the Yellow Players and Ferroptosis Therapeutic Strategies

**Figure 4:**
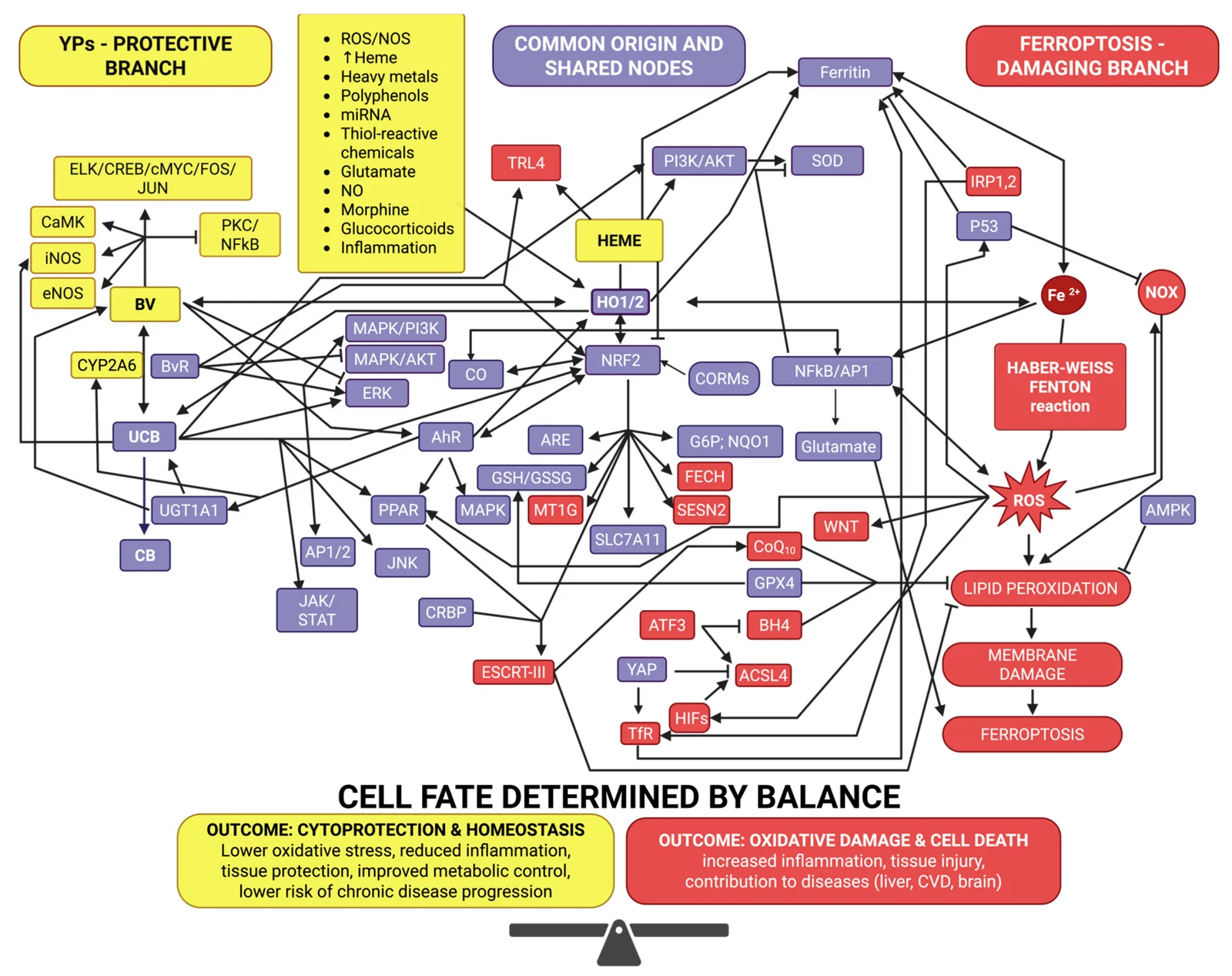
Interplay between ferroptosis and YPs. Yellow boxes: yellow players; red boxes: ferroptosis players; violet boxes: common players between YP and ferroptosis; one-way arrow: effect only in one direction; two-way arrows: effects on both directions. Fe^2+^: ferrous iron; BV: biliverdin; BVR: biliverdin reductase; UCB: unconjugated bilirubin; UGT1A1: uridine diphosphate glucuronosyltransferase; CB: conjugated bilirubin; ROS: reactive oxygen species; CYP: cytochrome P450 protein; TfR: transferrin receptor; GSH/GSSG: reduced/oxidized glutathione; GPX4: glutathione peroxidase 4; CO: carbon monoxide; NFkB: nuclear factor kappa-light-chain-enhancer of activated B cells; PPAR: peroxisome proliferator-activated receptors; NRF2: nuclear factor erythroid 2-related factor 2; ATF: activating transcription factor; BH4: tetrahydrobiopterin; p53: tumor protein p53; IRP: iron-regulatory protein; AMPK: 5′-adenosine monophosphate-activated protein kinase; ACSL4: acyl-CoA synthetase long chain family member 4; HIF: hypoxia-inducible factor; YAP: yes-associated protein 1; NOX: NADPH oxidase; H_2_O_2_: hydrogen peroxide; OH: hydroxyl group; OH^•^: hydroxyl radical; G6P: glucose-6 phosphate; ESCRT III: endosomal sorting complex required for transport III; CRBP: cellular retinol-binding protein; CoQ_10_: coenzyme Q_10_; WNT: wingless; STEAP3: six-transmembrane epithelial antigen of the prostate 3; NQO1: NAD(P)H dehydrogenase [quinone] 1; MT1G: metallothionein 1G; GSS: glutathione synthetase; FECH: ferrochelatase; SESN2: sestrin 2; MTOR: mechanistic target of rapamycin kinase; SOD: superoxide dismutase; miRNA: micro ribonucleic acid; NO: nitric oxide; PI3K: phosphoinositide 3-kinase; ERK: extracellular signal-regulated kinase; ELK: ETS-like protein 1; AhR: aryl hydrocarbon receptor; iNOS: inducible nitric oxide synthase; eNOS: endothelial nitric oxide synthase; CaMK: calcium/calmodulin-dependent protein kinase; JNK: c-Jun N-terminal kinase; AP1–2: adaptor protein 1–2; ATF: activating transcription factor; CREB: cAMP responsive element-binding protein 1; JAK: Janus kinase; STAT: signal transducer and activator of transcription; PKC: protein kinase C; AKT: serine/threonine kinase 1; cMYC: MYC proto-oncogene; FOS: FOS proto-oncogene; JUN: JUN proto-oncogene; MT1G: metallothionein 1G. Created in BioRender. Dalla Verde, C. (2026) https://BioRender.com/ysu4fq5.

Although our understanding of the deep and complex interplay between YPs and ferroptosis is still in its infancy, common nodes already emerge when considering the main molecules used to modulate these two opposing cellular processes (Figure 4).

**Figure 5:**
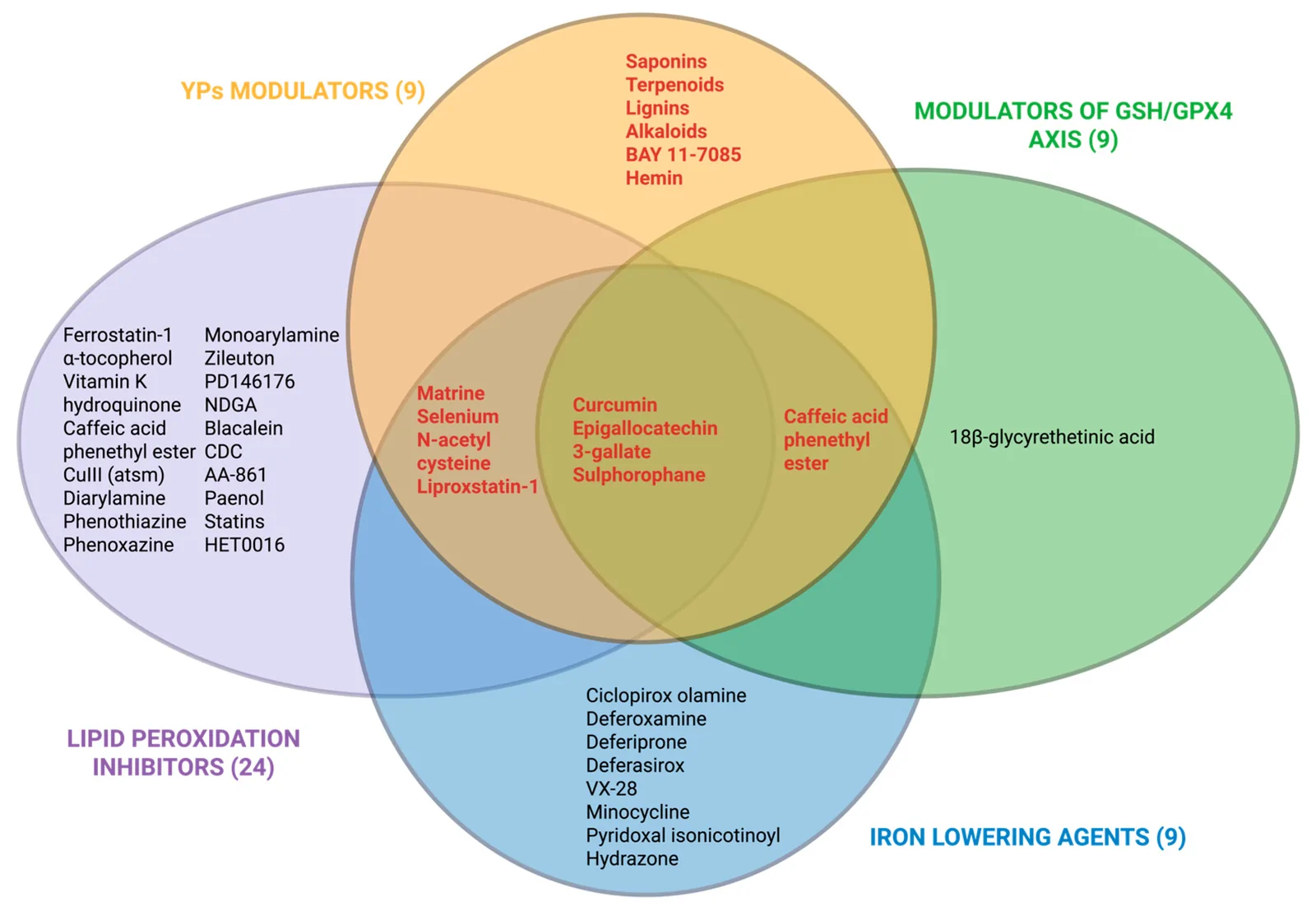
Venn diagram of the molecules and their targets. Created in BioRender. Dalla Verde, C. (2026) https://BioRender.com/kax118s.

Currently, the induction of YPs plays primarily through the upregulation of HO1, targeting the BV/UCB axis, or by using of tetrapyrrole- and heme-mimetic compounds [10,12,158,236,243,244,245,246,247,248,249]. Classic strategies to prevent or attenuate ferroptotic damage include: (1) reducing intracellular iron availability [224,225,226,227,228,229,230,231,232,233,234,235,236,237]; (2) inhibiting lipid peroxidation [243,244,250,251,252,253,254,255,256,257,258,259,260,261,262,263,264,265,266,267,268], and (3) modulating GSH/GPX4 antioxidant system [242,243,244,253,254,259,263,264,265,266,267,268]. As based on the Venn diagram in Figure 5, modulators of the YP pathway might not only affect single therapeutic targets on ferroptosis but also have the potential to simultaneously influence all three canonical regulators (iron metabolism, lipid peroxidation, and endogenous antioxidant defenses).

As examples, deferoxamine (DFO; Desferal) is a well-known ferroptosis inhibitor, primarily known for trapping iron. In addition, it can act as a downstream rescue factor interrupting ferroptotic execution even when the initiating lesion is a profound failure of antioxidant defenses (Xct, GPX4), independently from iron level [12,245,246]. Apparently, if this makes DFO/Deferral a powerful molecule in modulating ferroptosis, its clinical use must face multiple limitations. First, it is hydrophilic, with a high molecular weight, a limited circulation time, short half-life, and cellular uptake mostly by endocytosis [247]. Indeed, DFO removes only one third of labile iron, without any effect at increasing dosages [247], but may have side effects such as renal failure and granulocyte deficiency [246]. Molecules overstepping these limitations are under development and testing [246,248]. Notably, iron participates in major physiologic processes [249,269] that its chelation may disrupt. If this approach is beneficial in tumors, this is not the case in other conditions when the goal is cell survival. Thus DFO, despite being a promising approach in experimental models of ferroptosis, might be less efficient/safe in clinic, possibly strictly depending on the disease/cellular context [270]. Iron chelators may downregulate HO1, potentially limiting the beneficial effects mediated by YPs [271]. As an example, and only as a speculative matter, a phase-II, randomized, double-blind clinical trial conducted on newly diagnosed patients of PD, never administered with Levodopa, deferiprone (DFP) lowered iron levels but patient symptoms worsened over 36 weeks of treatment [272]. While this can be due to some toxic effect of iron chelators, speculatively it might be attributed to an imbalance between inhibiting ferroptosis and the reduced protection due to decreased YP’s activity [273].

As discussed in Section 3.3 and Section 3.4, ferroptosis may also act independently from iron excess, when the antioxidant defenses are overstepped. Supplementation of antioxidants (as example NAC) may foster the Cysteine/Xc-GSH-GPX4 trajectory and possibly directly affect the GPX4 reducing capacity countering lipid peroxidation [274]. FSP1 may counteract lipid peroxidation on a different level independently from GSH/GPX4 by regenerating CoQ10, a ROS trapping [157]. On this line (ROS trapping, independent from GSH/GPX4), this work also shows that ferrostatin 1 and liproxstatin 1 [12,275], as well as alpha-tocopherol [157], possibly synergically interact with BH4 [276] on the dihydrofolate reductase that catalyzes the regeneration of BH4. It is important to note that lipid-soluble bilirubin has the same antiferroptotic activities as lipophilic antioxidant enzymes, in particular vitamin A and E [268,277], and is possibly playing in synergy with tocopherols [1,149,150]. Mitochondrial membranes are protected from lipoperoxidation by a specific, GPX4-independent mechanism, comprising dihydroorotate dehydrogenase-mediated reduction of ubiquinone to ubiquinol, a radical-trapping antioxidant with anti-ferroptosis activity, at the inner mitochondrial membrane, in this way protecting an organelle fostering the Haber–Weiss–Fenton reaction [278]. In addition to what has been stated before, tocotrienols (belonging to the family of vitamin E) exhibit superior lipoperoxidation inhibition over tocopherols counteracting GPX4, xCT or glutathione synthesis inhibition [279]. All these mechanisms are relevant targets for pharmacologic modulation of ferroptosis. Importantly for the aims of this review, independently from the level, all these mechanisms work in protecting membranes, the final target of ferroptosis. In this respect bilirubin and the YPs may be viewed as components of a compensations strategy when the multilevel anti-ferroptotic systems collapse, conceptually working with CoQH_2_, BH4, tocopherols, radical-trapping antioxidant, and anti-lipoperoxidation as explained above [1,29,146,147,148,149,150,268,277,279].

In summary, even in the light of the current limited understanding, the overlap in the effects of pharmacological modulators targeting either pathway should not be regarded as unexpected. The complex network deeply interconnecting YPs and ferroptosis that is emerging suggests that future studies may need to move beyond investigating YPs and ferroptosis as separate mechanisms. Instead, experimental approaches should ideally monitor both systems simultaneously, quantitatively assessing the extent of activation or inhibition of each pathway and relating these changes to the resulting balance between cytoprotection and cell death. Importantly, such relationships are unlikely to be universal and will need to be defined in a disease-, tissue-, and possibly cell type-specific manner.

Among YP-targeted interventions, the strongest experimental evidence currently supports natural polyphenols and flavonoids, which exert potent antioxidant, anti-inflammatory, and cytoprotective effects, largely through activation of the HO1 pathway. Several synthetic HO1 modulators have also demonstrated promising antiferroptotic activity in preclinical models [166,174,280,281,282,283,284] (Figure 5).

Natural compounds are a valid alternative with the possibility to regulate multiple targets that have synergic anti-inflammatory and neuroprotective effects and can be used for long-term treatments since they have good safety and tolerability. Owing to their pleiotropic mechanisms of action, natural compounds can simultaneously modulate multiple pathways involved in ferroptosis, including oxidative stress, inflammation, iron homeostasis, and redox signaling, thereby providing synergistic cytoprotective and neuroprotective effects. In addition, their generally favorable safety and tolerability profiles make them attractive candidates for long-term treatment of chronic disorders. Other negative aspects are the low viability over time after administration and the variation at the batch level due to the lack of standardization in the production.

In contrast, synthetic small molecule ferroptosis modulators are designed to selectively target key components of the ferroptotic machinery. Their principal advantages include high target specificity, rapid pharmacological activity, and low IC50 in in vitro tests. Their clinical translation remains limited by several factors, including poor metabolic stability, potential off-target effects, and, particularly for neurological disorders, insufficient penetration of the blood–brain barrier (BBB) [166,174,280,281,282,283,284].

## 6. The Path from Research to Clinic

Bilirubin and the entire heme catabolic pathway have been shown in recent years to have an important biological significance, including modulation of many metabolic pathways. This review highlights how ferroptosis—one of the oldest and most evolutionarily conserved forms of cell death—and YPs with their emerging homeostatic and protective role against the most prevalent age-related diseases, are profoundly and intrinsically interconnected. This relationship extends far beyond iron, glutathione, and opposing effects on cell membranes, operating through an intricate network of signaling pathways. As the modulation of both mechanisms gains increasing traction as potential therapeutic targets, this high level of connectivity must be carefully evaluated empirically. This is particularly crucial given that the balance between these two players and their outcomes (death vs. adaptation and resistance) is highly tissue- and disease-dependent. Consequently, the development of future pharmaceuticals must also consider and balance the potential of targeted versus systemic delivery. Further research is needed to deeply understand the pathophysiologic equilibrium between YPs and ferroptosis, as well as its modulation in the context of each disease.

## 7. Limitations

We did not enter details on the specific markers of ferroptosis, assessment of the pharmacological effects on ferroptosis target components, and stability constants of specific iron chelators, as well as bioflavonoids, bilirubin, and thiol compounds.

## 8. Conclusions

Collectively, the available evidence suggests that pharmacological modulation of the YP pathway represents a particularly attractive therapeutic strategy because it integrates several fundamental mechanisms regulating ferroptosis. Through its effects on iron homeostasis, lipid peroxidation, glutathione metabolism, and inflammatory signaling, the YP pathway may provide broader cytoprotection than interventions directed at a single ferroptotic target. However, the dual role of HO1, which generates both cytoprotective bilirubin and potentially pro-ferroptotic ferrous iron, underscores the need for therapeutic approaches capable of enhancing the beneficial arm of the pathway while limiting iron-mediated toxicity. Further mechanistic studies and well-designed clinical trials are required to determine whether modulation of the YP pathway can be translated into effective therapies for ferroptosis-associated disorders.

## Data Availability

No new data were created or analyzed in this study. Data sharing is not applicable to this article.

## Abbreviations

The following abbreviations are used in this manuscript:

AA	Arachidonic acid
Aβ	β-amyloid
ACSL	Acyl-CoA synthetase long-chain family member
AD	Alzheimer’s disease
AhR	Aryl hydrocarbon receptor
AKT	Serine/threonine kinase 1
ALS	Amyotrophic lateral sclerosis
AMPK	AMP-activated protein kinase
AP1/2	Activator protein
ARE	Antioxidant Responsive Elements
ATF	Activating transcription factor
ATP	Adenosine triphosphate
ATG	Autophagy-related protein
BACE1	β-secretase 1
BAP1	BRCA1-associated protein 1
BBB	Blood–brain barrier
BECN	Beclin
BH2	Dihydrobiopterin
BH4	Tetrahydro biopterin
BV	Biliverdin
BVR	Biliverdin reductase
CaMK	Ca2+/calmodulin-dependent protein kinase
cAMP	Cyclic adenosine monophosphate
CARS1	Cysteinyl-tRNA synthetase 1
CB	Conjugated bilirubin
cMYC	MYC proto-oncogene
CO	Carbon monoxide
CoQ10	Coenzyme Q10
CoQ10H2	Ubiquinol
CORMs	CO-releasing molecules
CRBP	Cellular retinol-binding proteins
CRE	cAMP response element
CREB	cAMP response element-binding protein
CVD	Cardiovascular diseases
CYP	Cytochrome P450 protein
DA	Dopamine
DAMP	Damage-associated molecular pattern
DHA	Docosahexaenoic acid
DMT	Divalent metal transporter
DRE	DNA replication-related element
eNOS	Endothelial nitric oxide synthase
EAE	Experimental autoimmune encephalomyelitis, model of MS
ELK	ETS-like protein 1
EPA	Eicosatetraenoic acid
ERK	Extracellular signal-regulated kinase
ESCRT	Endosomal sorting complex required for transport
Fe^2+^	Ferrous iron
Fe^3+^	Ferric iron
FECH	Ferrochelatase
Fer-1	Ferrostatin-1
FOS	FOS proto-oncogene
FPN1	Ferroportin-1
FPP	Farnesyl pyrophosphate
FSP1	Ferroptosis suppressor protein 1 (initially referred to p53-responsive gene 3—PRG3; also known as AIMF2: apoptosis-inducing factor mitochondria-associated 2, or PRG3: p53-responsive gene 3)
FTH1	Ferritin heavy polypeptide 1
GGT	Gamma-glutamyl transferase
G6P	Glucose-6-phosphate
G6PDH	Glucose-6-phosphate dehydrogenase
GPX4	Glutathione peroxidase 4
GSH	Reduced glutathione
GSS	Glutathione synthetase
GSSG	Oxidized glutathione (Glutathione disulfide)
HCC	Hepatocellular carcinoma
HD	Huntington’s disease
HO	Heme oxygenase
HIFs	Hypoxia-inducible factors
iNOS	Inducible nitric oxide synthase
IPP	Isopentyl pyrophosphate
IRE	Iron-responsive elements
IRP	Iron-regulatory proteins
IS	Ischemic stroke
LDL	Low-density lipoprotein
JAK	Janus kinase
JNK	c-Jun N-terminal kinase
JUN	JUN proto-oncogene
KEAP1	Kelch-like ECH associated protein 1
MDA	Malondialdehyde
MAPK	Mitogen-activated protein kinase
MAFLD	Metabolic dysfunction-associated steatotic liver disease
MASH	Metabolic dysfunction-associated steatohepatitis
miRNA	Micro RNA
MARE	Maf recognition element
MS	Multiple sclerosis
MT1G	Metallothionein 1G
MUFAs	Monounsaturated fatty acids
mTOR	Mechanistic target of rapamycin kinase
NADPH/NADP	Reduced/oxidized nicotinamide adenine dinucleotide phosphate
NCOA4	Nuclear receptor coactivator 4
NFκB	Nuclear factor kappa B
NM	Neuro melanin
NO	Nitric oxide
NOS	Nitric oxide species
NOX	Nicotinamide adenine dinucleotide phosphate NADPH oxidase
NQO1	NAD(P)H quinone dehydrogenase 1
NRF2	Nuclear factor erythroid 2-related factor 2
OH^•^	Hydroxyl radical
O_2_^•−^	Superoxide
pRb	Phosphorylated retinoblastoma protein
PD	Parkinson’s disease
PGD	Phosphogluconate dehydrogenase
PI3K-Akt	Phosphatidylinositol-4,5-bisphosphate 3 kinase—protein kinase B
PKC	Protein kinase C
PPAR	Peroxisome proliferator-activated receptor
PPRE	Peroxisome proliferator response element
PXR	Pregnane X receptor
PTEN	Phosphatase and tensin homolog
PUFAs	Polyunsaturated fatty acids
R5P	Ribose 5-phosphate
RNS	Reactive nitrogen species
ROS	Reactive oxygen species
SCD	Stearoyl-CoA desaturase
SESN2	Sestrin 2
SLC	Solute carrier protein
SOD	Superoxide dismutase
SREBP1	Sterol regulatory element-binding protein 1
STAT	Signal transducers and activators of transcription
STEAP3	Six-transmembrane epithelial antigen of the prostate 3
STING1	Stimulator of interferon response CGAMP interactor 1 (also known as TMEM173)
TFH	Ferritin heavy polypeptide
TfR	Transferrin receptor
TGF	Transforming growth factor
TLR	Toll-like receptor
TP53/p53	Transcription factor tumor protein
UCB	Unconjugated bilirubin
UGT1A1	Uridine diphosphate glucuronosyltransferase
Xc system	Cystine-glutamate transport system
YPs	Yellow players
WNT	Wingless-related integration site signaling pathway
XRE	Xenobiotic response element
YAP	Yes-associated protein 1
